# Leveraging TikTok in Plastic Surgery: A Practical Framework for Patient Education, Professional Branding, and Ethical Use

**DOI:** 10.1093/asjof/ojag084

**Published:** 2026-05-12

**Authors:** Sakar Gupta, Nada E Botros, Alisha S Khosla, Emily E Zona, Keenan S Fine, Peter J Wirth, Venkat K Rao

## Abstract

TikTok (Culver City, CA), with over 1.6 billion active monthly users, has emerged as an ideal social media platform for plastic surgeons. With its unique audiovisual format, personalized algorithm, and broad audience, TikTok has become an increasingly effective medium for surgeons to interact with patients, share educational content, and build professional brands. However, despite its popularity, many surgeons, especially those who established their practice before this digital landscape, might be unfamiliar with how to effectively utilize the platform and leverage its unique features. This paper provides a comprehensive practical guide for plastic surgeons to strategically design, optimize, and maintain a TikTok presence. We describe key aspects of the platform, including its unique For You Page (FYP) algorithm and demographic distribution. Furthermore, we offer insights on profile optimization, content strategy, actively analyzing content performance, and ethical considerations relevant to social media use. When used strategically, TikTok can serve as an effective tool for patient education, audience engagement, and maintaining professional visibility within plastic surgery.

Level of Evidence: 5 (Therapeutic)

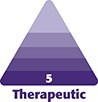

Social media has transformed the way surgeons communicate with patients.^1^ It has emerged as one of the most popular and effective tools for education, patient engagement, and professional outreach.^1^ Plastic surgeons have increasingly utilized social media as an avenue for direct-to-consumer marketing, with >92% reporting using social media for advertising as compared with just 30% in 2010.^2^ This profound increase reflects a dramatic shift in the patient's decision-making process. Patients, especially younger adults, are strongly influenced by social media and, as a result, are turning to online platforms as their primary means for procedure education, surgeon research, peer reviews, and expectations for treatment outcomes.^2^ According to a recent analysis of women undergoing breast augmentation, 97% of Millennials and 92% of Generation X reported using social media platforms compared with 0% of Baby Boomers. Among them, 64% of the women reported relying on social media to guide their decisions regarding the procedure.^3^

With over 1.6 billion active monthly users, TikTok (Culver City, CA) has become one of the most popular social media platforms, trailing just behind Instagram (2 billion active monthly users globally).^4^ Compared with Instagram, however, content related to plastic surgery on TikTok generates significantly higher engagement (total number of likes, views, and comments), with an average of 438,261 per post compared with Instagram's 275,565.^1^ Educational content on TikTok generates remarkably high levels of user engagement, averaging 448,490 interactions per post.^1^ The expanded visibility can be largely attributed to TikTok's personalized “For You Page” (FYP) algorithm, which enables users without large follower bases to reach a wide audience. Armed with its unique audiovisual interface, TikTok has garnered rapid popularity among younger social media users who gravitate more toward interactive and engaging audiovisual content.^5^

Plastic surgeons around the world have been increasingly capitalizing on TikTok's short-form audiovisual content to reach new patients and strengthen their professional network.^5^ Yet, many surgeons, such as those who established their practice before this digital landscape, may be less familiar with how to harness this tool adequately. Given the importance of branding and visibility in modern plastic surgery, this paper aims to provide a practical guide for plastic surgeons to optimize the use of TikTok in their practice.

## UNDERSTANDING TIKTOK

### Overview of TikTok as a Platform

TikTok is a short-video social media platform strategically designed for creating, sharing, and discovering short videos. Its proprietary algorithm is centered around the “FYP,” which is a personalized, evolving feed that changes based on the user's interests and preferences over time.^6^ The algorithm integrates user engagement metrics, such as watch time, shares, comments, saves, follows, and likes, as primary inputs for its recommendation engine.^6,7^ The system constantly adapts, amplifying content that shows greater user engagement (likes, saves, follows, etc.) and filtering out content with negative signals. Video features such as keywords, trending audio clips, and popular visual effects further help to produce a dynamic and unique experience for each user.^6,7^ The more active the user is on the app, the more the algorithm learns about their interests, and the better it can predict what they want to see. The result is an application where no 2 TikTok users see the same combination of videos on their FYP.^6,7^

### Demographic Patterns

Gen Z and Millennials dominate TikTok's user base. In the United States, individuals aged 18 to 34 represent the majority of active users.^8^ In a recent Pew Research Center study, the authors demonstrate that usage among US adults is highest for those aged 18 to 29 (59%), compared with 40% of those aged 30 to 49 and only 10% of adults 65 and older.^9^ Recently, there has also been an increase in the prevalence of healthcare professionals on the platform. As of 2021, there were over 3.8 million healthcare content creators on TikTok. In 2023 alone, users spent an average of 630 million hours watching videos with the hashtag #Doctors, and hashtags such as #HealthTips garnered over 11.2 billion views.^10^ Interestingly, 1 in 5 Americans say they trust health influencers more than medical professionals in their community and turn to TikTok before their doctor when seeking treatment for a health condition.^10^ These statistics highlight TikTok's influence in disseminating medical information and connecting with patients.

As a result, TikTok serves as an ideal platform for new and established plastic surgeons to build and expand their network. The algorithm can effectively and efficiently expand the creator's reach with minimal previous online connections. New plastic surgeons can interact directly with patients beyond their follower base if their content aligns with the viewer's interests and search patterns. Among the broad landscape of social media platforms, TikTok offers a unique opportunity for rapid visibility and growth.^7,10^

### Building a Professional TikTok Profile

A TikTok profile is the user's first impression of the plastic surgeon. Hence, it is essential to create a well-designed and appealing profile to effectively showcase the surgeon's expertise, establish credibility, and encourage new users to follow the account.

#### Choosing a Profile Name

Choose a username that is professional, yet easy to remember. Long and descriptive names can be distracting and hard to quickly recognize. Tailoring usernames to reflect the plastic surgeons’ specialty practice (for instance, @CosmeticDr.M) can be an effective way to gain attention.^10,11^ It is also important to ensure that the username is easy to search for. Avoiding special characters or numbers can improve searchability. Furthermore, consistent branding can help bridge a surgeon's audience across social media platforms.^10,11^

#### Profile Photograph and Bio Description

A clean and easy-to-recognize, high-quality, professional photograph can help establish credibility and trust from the beginning.^11^ Surgeons can even use their clinic or practice logo because their profile image if they have an established brand. Some creators recommend curating the profile in a way that conveys the personality of the surgeon's brand.^11,12^ For example, an aesthetic surgeon might choose a soft, stylized logo that signals artistry and approachability.

A bio is a concise profile description that can help educate potential viewers of the surgeon's identity, specialty, and breadth of offered services.^12^ Using short phrases to clearly describe the specialty and type of content is a key to crafting an effective bio. For example, surgeons with distinct expertise in technical skills or those who offer unique procedures can choose to highlight these features in their bio, pairing them with call-to-action prompts that invite the user to learn more or book an appointment.^12^ This can help further attract niche audiences actively seeking those services. In addition, TikTok permits users with a registered business account or with 1000 or more followers to add a link to their profile.^13^ Surgeons can use this feature to share a link to their website or consultation services. A good strategy for directing users to multiple links, however, is to create a link-in-bio landing page (eg, Linktree), which allows the surgeon to aggregate several URLs and resources under a consolidated profile link.

#### Pinned Videos

TikTok allows users to pin 3 videos at the top of their profile. First impressions are often built within seconds, which is why strategically pinning videos can serve as another highly effective strategy to create a lasting impression on the user. Content such as introductory profiles, specialty procedures, or impressive before-and-after images are a few ways to educate and engage potential patients.^14^ Furthermore, pinning is also a useful tool to help prevent important content from getting lost and ensure that those videos are readily accessible to new users.

### Content Strategy for a Plastic Surgeon's TikTok Account

An effective content strategy is essential for plastic surgeons to engage their target audience and maintain relevance in this crowded platform. A successful content strategy that resonates on social media must prioritize storytelling rather than communication through facts and statistics.^15^ Plastic surgeons who have utilized TikTok report that storytelling through patient journeys and experiences helps make the content more relatable and memorable, allowing the surgeon to better connect with their audience and capture their attention.^15^ Regular engagement with the audience through comments and messages can further help build an online community. Surgeons have reported that these active interactions with users are an effective way to foster loyalty, increase word-of-mouth referrals, and retain followers.^15^ Plastic surgeons can manifest these engagement principles through a range of content formats on TikTok. Here, we highlight the use of a combination of educational, transformational, nonmedical, and trend-based categories to form a comprehensive content strategy.

#### Educational Content

Educational videos are at the forefront of content creation for plastic surgeons.^15^ In a recent study, Patel et al identified educational content as one of the greatest drivers of engagement and follower growth among plastic surgeons on TikTok, averaging 448,490 engagement per post.^1^ These videos, which include explanations of plastic surgery procedures, surgery techniques, recovery times, and outcomes, can help build credibility and inform potential patients.^15^ Surgeons can create short videos using infographics or animations to simplify and portray common procedures. Furthermore, content about postoperative care tips and dispelling common myths and misconceptions about the field carries favorable “viral” potential across the platform's young demographic.

#### Transformation Content

Given that TikTok is a platform driven by visual content, it lends itself well to sharing transformational before-and-after content. This includes any content that depicts preoperative and postoperative surgical changes in a patient, including before-and-after photographs and healing timelines (eg, “Day 1→Week 2→1 month”).^1^ The significant appeal behind transformation posts lies in their ability to provide patients with a better understanding of the expected outcomes while simultaneously serving as a powerful showcase of the surgeon's skills and results.^15^ This result-driven approach can build the surgeon's trust and credibility and can generate significant engagement on the platform.^1,15^

#### Nonmedical Content

Authenticity and trust are keys to establish longevity and sustainability on the platform. Plastic surgeons on TikTok emphasize the importance of creating a bond between the surgeon and potential patients.^15^ Sharing content depicting the surgeon's day-to-day activities, personal life, values, and culture of their practice can help nurture this bond and humanize the surgeon.^15^ This strategy provides a unique character to a professional account, which can help separate it from the rest of the competition. Furthermore, nonmedical content also includes teaching videos such as study tips and board examination preparation strategies. In a review of a single plastic surgeon's experience on TikTok, videos aimed at such education received the highest engagement and views.^15^ These content strategies can be used to establish a strong following that can then later be exposed to more targeted surgeon-specific content.^3^

#### Leveraging Trends

TikTok thrives on trends—popular sounds and hashtags are among its defining features.^16^ Incorporating trending elements, audio, or music into videos can increase the likelihood that the content is seen by a wider audience.^16^ Leveraging these trends also allows a surgeon to readily join active conversations by aligning content with popular search items. For instance, plastic surgeons can pair explanations of surgeries or reaction videos to trending graphics or sounds, which can boost their content discoverability. Furthermore, commentary on plastic surgery trends within popular culture represents another powerful engagement tool. Studies have demonstrated that videos regarding celebrity-associated plastic surgery trends garner substantial online traffic because of their cultural relevance, with average interactions approaching 1 million per post on Instagram and over 200,000 per post on TikTok.^1^ Therefore, for plastic surgeons entering the TikTok landscape, carefully framed videos that target popular celebrity-related trends—without speculating about individual patients—can carry meaningful “viral” potential while still emphasizing professionalism and education.^1^

### Analyzing Profile Data on TikTok

As the plastic surgeon builds their TikTok presence, it is imperative to continue to routinely reflect on current content strategies in order to understand what type of content best resonates with the target audience.^17^ TikTok's “analytics” tool provides comprehensive data and performance metrics that allow users to evaluate the effectiveness of their current content and engagement strategies.^17^ These insights can then be used to actively adapt future content to better align with the surgeon's target demographic. Here, we summarize some of the key analytical tools that a plastic surgeon may choose to use to actively audit their content strategy (Table 1).

**Table 1.** Summary of Key TikTok Analytics Tools and Their Relevance in Plastic Surgery

Analytic category	Description	Key metrics	Utility for plastic surgeons
Audience interaction	Measures how users engage with a creator's content	Likes, views, shares, comments, traffic source, search queries	Identifies which content types (educational, marketing, and lifestyle) most effectively drive engagement. Understanding traffic sources (eg, For You Page vs search) and search terms (eg, “rhinoplasty recovery”) helps tailor future posts
Follower data	Details demographic and content consumption patterns of followers	Follower growth per post, gender distribution, age range, geographic location, active hours	Enables surgeons to understand their target audience's identity and preferences, optimize posting schedules, and adapt messaging tone (eg, educational vs marketing content) for specific demographics
Viewer data	Provides aggregate insights into both followers and nonfollowers who watch content	Unique viewers, returning viewers, inferred demographics, most active times	Helps differentiate audience loyalty vs reach to new users. Timing insights guide optimal posting schedules. Enables understanding of broader appeal beyond established follower base
Video performance	Evaluates engagement dynamics within individual videos	Retention rate, average watch time, drop-off points, completion rate	Identifies segments within videos where viewer engagement declines, enabling refinement of pacing, introductory hooks, and clarity of educational messaging

#### Audience Interaction

TikTok's algorithm rewards content that generates more engagement. Surgeons can use metrics such as Likes, Views, Shares, and Comments to readily gauge the audience's level of interaction with their content.^15,17^ Stratifying these metrics by individual videos or different content categories can allow a surgeon to obtain direct feedback about the type of content that generates the greatest engagement.^15^ Furthermore, surgeons can use the “Traffic source” and “Search queries” data to determine how viewers are actively discovering their content (FYP, search, hashtag pages, sound pages, etc) and what search terms (eg, “cleft lip surgery,” “Botox tips,” etc) lead them to the surgeon's posts.^17^

#### Followers’ Data

A detailed understanding of the surgeon's current follower base is a key to building a successful online presence.^15^ Surgeons can assess follower growth by tracking the number of new followers acquired with each post. They can also analyze the gender distribution, age ranges, and geographic locations of their followers and explore their general content consumption habits and timing.^15,17^ Plastic surgeons can leverage this information to understand who exactly is consuming their content and when during the day or week they are most likely to engage.

#### Viewers Data

The “Viewers” tool provides a comprehensive overview of the user's overall audience—not just followers. It helps the surgeon understand who their content is appealing to.^17^ TikTok uses predictive models rather than confirmed user data to provide insights into inferred demographic information of viewers, including age, gender, and location. Key metrics regarding the number of “Unique viewers” (how many different people watch the videos) and “Returning viewers” (how many people come back to watch the videos again) can help a surgeon differentiate whether their content is building loyalty or mostly reaching new audiences.^15,17^ Furthermore, activity data (“Most Active Times”) can be used to determine the ideal times to post based on when viewers are most active on the app.^17^

#### Video Performance Analytics

TikTok offers unique individual video analytic features that can help optimize content performance. For instance, “Retention rate” is described as the percentage of the video watched by the user, and “Average watch time” is the average duration viewers watched the video.^15,17^ Surgeons can leverage these tools to find exact time points in the video where most users liked the post or stopped watching. These metrics can be used to help determine specific elements within videos that encourage engagement vs those that lead to a decrease in interest.

### Ethical Considerations

Although TikTok offers many opportunities for plastic surgeons, various ethical considerations must be considered to ensure that the published content adheres to medical and ethical standards that uphold the trust and integrity of our profession. Because TikTok prioritizes surgeons based on their follower count and popularity instead of academic merit, the line between professional medical advice and misinformation can easily blur.^15^ The unregulated nature and limited oversight of these platforms can further disseminate misinformation and have significant consequences for patient safety.^15^

Plastic surgeons on TikTok should adhere to the American Society of Plastic Surgeons Code of Ethics to guide their social media engagement. Some pertinent considerations are described below.^15,18-20^

#### Patient Privacy and Consent

Surgeons need to obtain explicit written informed consent before posting any patient images or content, as they “may not reveal a patient's confidence…or an information obtained from the patient in a professional capacity without such patient's consent…”^19,20^ Protecting patient confidentiality and privacy follows all legal requirements, including the Health Insurance Portability and Accountability Act of 1996.^20^

#### Professionalism and Trust

Surgeons must uphold the dignity of the profession by avoiding misleading information or unprofessional conduct that can harm the patient and the specialty. It is our responsibility to ensure that public communications are not “false, fraudulent, deceptive, or misleading.”^19^

#### Honest and Transparent Advertisement

Advertisements should not use communication that is “likely to create false or unjustified expectations of favorable results.”^19^ Surgeons should “strive to use accurate and respectful language and images” and “expose, without hesitation, illegal or unethical conduct of fellow members of the profession.”^19^ Furthermore, surgeons should remain cautious when using beauty filters or digital enhancement tools in their content. Unlabeled or excessive modification of images may misrepresent surgical outcomes, contribute to unrealistic patient expectations, and risk violating ethical standards regarding truthful and transparent advertising.

#### Compensation Disclosures and Marketing

Surgeons should disclose all compensations for testimonials or endorsements related to medical care or qualifications in order to “permit the free and complete exercise of sound medical judgment and skill.”^19^ The Code of Ethics prevents members’ communications from containing “endorsement pertaining to the quality of the member's medical care…if the endorser has been compensated by the Member…for making such testimonial or endorsement.”^19^ Offering free or discounted services in exchange for online publicity without proper disclosure can blur ethical and legal lines, risking the compromise of professional integrity.^19^

## CONCLUSIONS

Because social media continues to define patient expectations and healthcare decisions, building a strategic online presence on platforms like TikTok can help plastic surgeons communicate efficiently, build trust, and maintain professional visibility in the digital landscape. However, building and maintaining a successful TikTok profile requires more than just passive participation alone; it requires a strong understanding of the platform's framework, an effective content strategy, and active performance analysis. Plastic surgeons can utilize the framework outlined in this paper to effectively navigate the underlying platform mechanics, content strategy, and ethical considerations behind developing a professional and sustainable presence on TikTok.
